# The lactylation axis: bridging metabolic reprogramming and immunosuppression in the tumor microenvironment

**DOI:** 10.3389/fimmu.2026.1743101

**Published:** 2026-06-19

**Authors:** Xingxing Tao, Yingjie Liu, Peishan Yu, Wenjiang Wu, Yanli Fu, Ping Lan, Shengzhu Jin

**Affiliations:** Department of Oncology, Shenzhen Hospital (Futian) of Guangzhou University of Chinese Medicine, Shenzhen, Guangdong, China

**Keywords:** protein lactylation, tumor microenvironment, metabolic reprogramming, immune evasion, epigenetics, cancer immunotherapy

## Abstract

Protein lactylation, a novel post-translational modification (lysine lactylation, Kla) driven by the oncometabolite lactate, has emerged as a critical epigenetic mechanism that directly links cellular metabolic state to gene regulation. Within the tumor microenvironment (TME), lactate accumulation resulting from the Warburg effect provides abundant substrate for lactylation, positioning this modification as a central hub in cancer biology. This review systematically elucidates the dual role of lactylation in driving tumor progression. Intrinsically, lactylation promotes tumor cell malignancy by globally reshaping chromatin accessibility via histone modifications (e.g., H3K18la) and orchestrating oncogenic signaling pathways through non-histone protein modifications, thereby enhancing metabolic reprogramming, proliferation, invasion, and therapy resistance. Extrinsically, lactylation serves as a key immunosuppressive mechanism by reprogramming the function of immune cells within the TME. It drives macrophages toward an M2-like immunosuppressive phenotype, enhances the suppressive function of regulatory T cells (Tregs), and induces dysfunction and exhaustion in CD8+ T cells, collectively fostering an immune-privileged niche. We further discuss the promising therapeutic strategies targeting the lactylation axis, including inhibitors of lactate production or lactyltransferases, and their combination with immune checkpoint blockade, to reverse immunosuppression and overcome treatment resistance. In summary, understanding the lactylation axis establishes a novel metabolic-epigenetic-immune paradigm and suggests potential new frameworks for precision cancer therapy.

## 1 Introduction

Protein post-translational modifications (PTMs) are fundamental mechanisms that regulate protein function, localization, and interactions, playing pivotal roles in tumorigenesis. Although classical PTMs, including phosphorylation, acetylation, and ubiquitination, have been extensively characterized for their roles in driving oncogenic signaling, metabolic reprogramming, and epigenetic remodeling (1), recent advances have unveiled a novel PTM— protein lactylation—that is rapidly emerging as a frontier in cancer research, particularly at the intersection of metabolism and immunity.

Traditionally viewed as a metabolic waste product of the Warburg effect, lactate accumulation contributes to an acidic and immunosuppressive tumor microenvironment (TME) (2, 3). However, a paradigm shift was catalyzed by the landmark discovery by Zhang et al. (2019) in *Nature*, which identified histone lysine lactylation (Kla) as a direct epigenetic modification linking lactate metabolism to gene expression (4). This finding redefined lactate from a mere metabolic byproduct to a key signaling molecule that serves as a substrate for PTMs, specifically lysine lactylation (hereafter referred to as lactylation or Kla). Lactylation, therefore, represents a direct molecular bridge between metabolic reprogramming and transcriptional regulation.

The unique conditions of the TME—characterized by hypoxia, acidosis, and immunosuppression—provide an ideal context for lactylation to exert its multifaceted functions. Subsequent research has expanded beyond histones, revealing that lactylation regulates a wide array of non-histone proteins, thereby influencing diverse processes from tumor cell proliferation and invasion to immune cell dysfunction. This positions lactylation as a central axis coordinating the crosstalk between cancer cells and immune cells within the TME.

This review aims to systematically delineate the role of the lactylation axis in shaping tumor progression. We will dissect its impact on metabolic reprogramming, tumor proliferation and migration, and, most importantly, immune evasion within the TME. Finally, we discuss the therapeutic potential of targeting lactylation, thereby integrating metabolic, epigenetic, and immune perspectives for future cancer precision therapy.

A note on causality. Current evidence is largely correlative. Causal strategies—site-specific lactylation mutations, conditional knock-in models, and bioorthogonal probes—are needed to dissect direct lactylation effects from lactate’s metabolic functions.

Technical limitations and emerging detection methods. panantiKla antibodies, while widely used, have limited specificity (cross-reactivity with other acyl modifications) and sensitivity (low stoichiometry of lactylation). To address these issues, advanced methods are emerging: lactylation-specific proteomics (pan-Kla antibody enrichment combined with LC-MS/MS) enables large-scale site identification, and bioorthogonal chemical probes (e.g., the alkynyl-lactate analogue YnLac) allow live-cell metabolic labeling and detection of lactylated proteins without antibody-based enrichment. These orthogonal tools help circumvent antibody-related limitations, though challenges remain in distinguishing L/D-lactate stereoisomers and developing robust quantitative assays.

## 2 Lactylation modification in the tumor microenvironment: a crossroads of metabolic remodeling and epigenetic regulation

**Figure 1:**
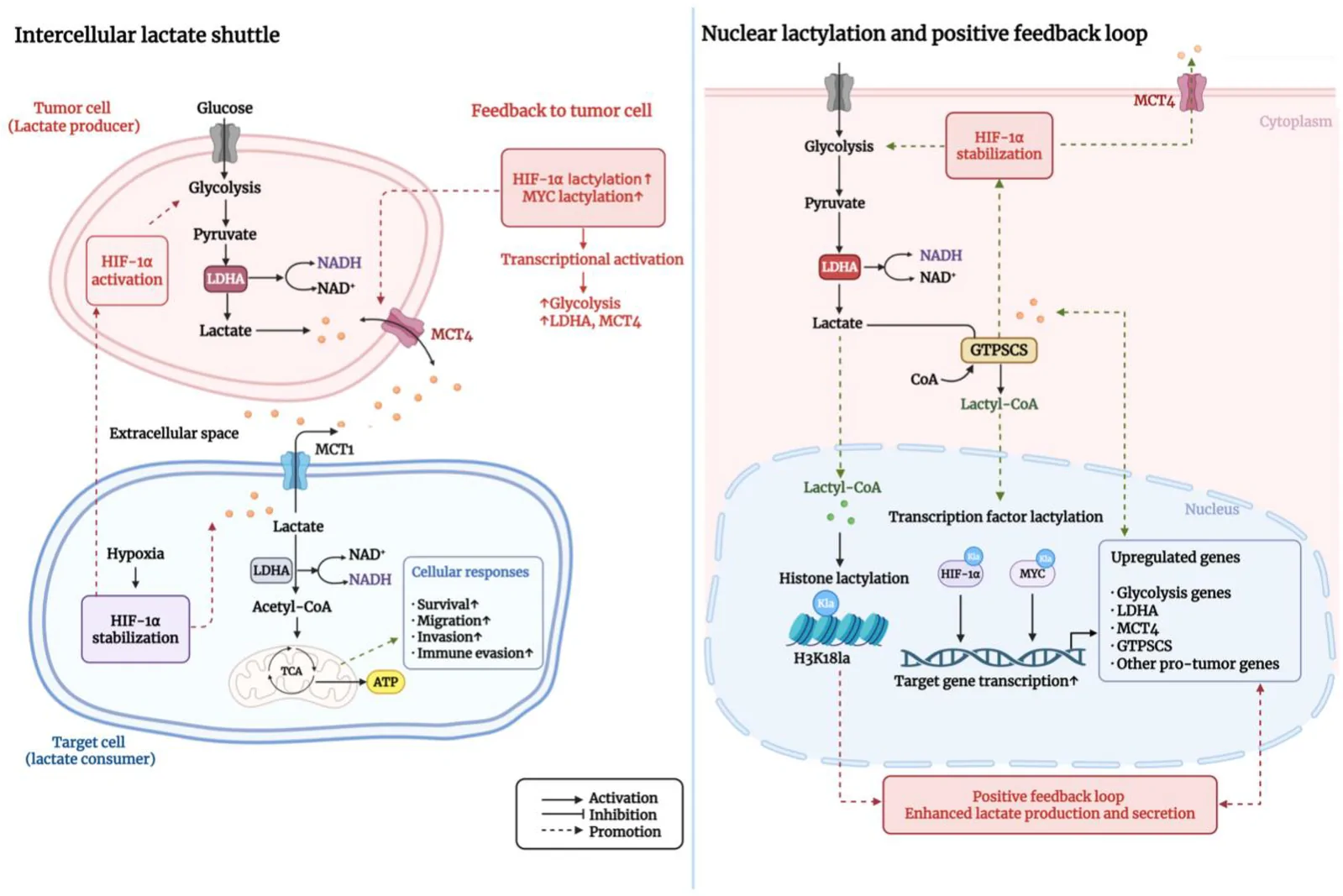
Lactate shuttling and protein lactylation in the tumor microenvironment. **(A)** Intercellular lactate shuttle. In hypoxic tumor cells, glycolysis is activated (e.g., via HIF-1α stabilization and LDHA/LDHB), producing large amounts of lactate. Tumor cells export lactate through MCT4, and extracellular lactate is taken up by neighboring target cells (e.g., immune cells, cancer-associated fibroblasts) via MCT1. Once inside the target cell, lactate can enter the mitochondria, be converted to acetyl-CoA, and fuel the TCA cycle, thereby supporting oxidative metabolism. **(B)** Nuclear lactylation and positive feedback loop. In the nucleus, lactate is converted to lactyl-CoA (e.g., by GTPSCS) and used by lactyltransferases such as p300 to catalyze histone lactylation (e.g., H3K18la) as well as lactylation of transcription factors (e.g., HIF-1α, MYC). Histone lactylation opens chromatin and activates transcription of glycolytic genes (e.g., LDHA, MCT4), while lactylated transcription factors further enhance glycolysis and lactate production. This creates a positive feedback loop that amplifies lactate generation, lactate secretion, and global lactylation, thereby sustaining the pro-tumorigenic metabolic-epigenetic network. Created in BioRender. Yu, P. (2026). HYPERLINK "https://biorender.com/idq9h42"https://BioRender.com/idq9h42.

The tumor microenvironment (TME) is a dynamic ecosystem composed of tumor cells, immune cells, stromal cells, the extracellular matrix (ECM), and physicochemical features such as hypoxia and acidosis. The formation of an acidic environment is a hallmark of the TME: tumor cells hyperactivate glycolysis via the Warburg effect, leading to massive lactate accumulation and a significant drop in extracellular pH (6.5–6.9). This acidic milieu not only drives tumor invasion and immune evasion through metabolic reprogramming (2, 5) but also facilitates epigenetic regulation via lactylation modification. Lactate shuttling between cell types in the TME (**Figure 1**). Extracellular lactate derived from glycolytic tumor cells does not remain static; it is actively transported across the TME via monocarboxylate transporters (MCTs). tumor cells often upregulate MCT4 for lactate export, while some express MCT1 for lactate import, whereas adjacent cells such as cancer-associated fibroblasts (CAFs), immune cells (e.g., macrophages, T cells), and even tumor cells themselves express MCT1 for lactate import. This heterotypic lactate exchange establishes metabolic symbiosis: imported lactate can be converted back to pyruvate and utilized for mitochondrial respiration, preserving glucose for glycolytic cells. Moreover, internalized lactate serves as a substrate for nuclear lactylation, thereby coupling intercellular metabolic cooperation to epigenetic regulation. Thus, the destination and diffusion of lactate within the TME—whether taken up by immune cells to drive lactylation-dependent immunosuppression or consumed by stromal cells for energy—critically shape the cell-type-specific lactylation landscape.

Lactate acts as a signaling molecule and is covalently attached to lysine residues on proteins by specific enzymes, forming lactyllysine (Kla), which directly modulates target protein function (4). This modification is uniquely coupled to cellular metabolism: the lactate concentration gradient within the TME dynamically drives lactylation levels, creating a direct link between metabolic status and processes such as chromatin remodeling and signal pathway activation (6).

Within the TME, lactylation promotes tumor progression through a multi-dimensional interactive network. In tumor cells, lactylation targets glycolytic enzymes (e.g., LDHA) and EMT-related transcription factors (e.g., Snail), establishing a positive feedback loop that fuels glycolysis and metastasis (7). In immune cells, lactylation suppresses IFN-γ secretion in cytotoxic T lymphocytes (CTLs) while enhancing the immunosuppressive function of regulatory T cells (Tregs), thereby fostering an immune-tolerant niche (8). In stromal cells, for instance, cancer-associated fibroblasts (CAFs) in pancreatic cancer enhance glycolysis to produce excess lactate, which is taken up by tumor cells, leading to histone H3K18 lactylation and subsequent activation of pro-neural invasion genes (9). Notably, the regulatory network of lactylation is not reliant on single protein targets but involves a synergistic effect between histone and non-histone lactylation. Histone lactylation (e.g., H3K18la) can globally reshape chromatin accessibility, while non-histone lactylation (e.g., on STAT1, PKM2) specifically regulates cellular behaviors through signaling cascades. Spatially and temporally coupled, these modifications collectively drive the malignant evolution of the TME. Nevertheless, a critical biochemical question arises regarding the specificity of p300/CBP as lactyltransferases. p300 is fundamentally a histone acetyltransferase that utilizes acetyl-CoA as its canonical substrate. However, within the lactate-rich TME, lactyl-CoA concentrations can reach levels that compete with acetyl-CoA. The molecular basis for p300’s preference for lactyl-CoA over acetyl-CoA at specific lysine residues—or conversely, its ability to use both promiscuously—remains incompletely understood. Emerging evidence suggests that the local concentrations of these two acyl-CoAs (10, 11)and their binding affinities to the p300 active site (12, 13) may collectively determine the enzymatic outcome. Moreover, the structural flexibility of p300 may permit induced-fit accommodation of the lactyl group. A deeper discussion of these competing substrates (lactyl-CoA vs. acetyl-CoA) and their fluctuating ratios in the TME would provide a more rigorous biochemical framework for understanding how lactylation is quantitatively regulated. Future structural and kinetic studies are urgently needed to resolve this specificity paradox.

Protein lactylation: synergistic effects between histone and non-histone modifications

As an emerging post-translational modification, protein lactylation exhibits dynamic synergistic effects between histone and non-histone modifications, playing critical roles in DNA repair, gene expression regulation, cellular metabolism, and immune responses.

In the field of DNA repair, histone lactylation facilitates the access of repair proteins (e.g., MRE11, NBS1) to damage sites by loosening chromatin structure (14). Meanwhile, non-histone lactylation directly modulates the activity of signaling pathways and metabolic enzymes. For instance, lactylation at the K673 site of MRE11 enhances its DNA-binding capacity (15), and lactylation of NBS1 at lysine 388 (K388) is crucial for the formation of the MRE11-RAD50-NBS1 (MRN) complex and the accumulation of homologous recombination (HR) repair proteins at DNA double-strand break sites (16). Thus, histone and non-histone lactylation act in concert to improve the efficiency of DNA end resection and homologous recombination, ensuring proficient DNA repair.

Regarding gene expression regulation, histone lactylation influences gene expression by altering chromosome architecture, modulating transcription factor binding, and regulating the accessibility of gene promoter regions. For example, histone H3K18 lactylation marks active promoters and enhancers, thereby increasing chromatin accessibility (17). In contrast, lactylation of non-histone proteins, such as Snail1, activates downstream pathways through nuclear translocation (18), establishing a dual-layered regulatory network encompassing both epigenetic modifications and transcription factor activity.

In metabolic control, histone H4K12 lactylation activates the transcription of glycolytic genes, enhancing glycolytic flux and lactate production (19) Conversely, lactylation at the K147 site of the non-histone protein aldolase A (Aldoa) dynamically fine-tunes glycolytic flux by inhibiting its enzymatic activity (20), enabling maintenance of metabolic homeostasis.

Within immune responses, histone lactylation drives the expression of inflammatory factors in macrophages (21), while non-histone lactylation modulates the intensity of innate immunity by regulating the cGAS-STING pathway (22). Together, they coordinately shape the direction and efficacy of the immune response.

This multi-layered synergy between histone and non-histone lactylation not only expands the functional scope of lactylation modifications but also provides a theoretical foundation for developing therapeutic strategies that target the metabolism-epigenetics interaction network.

Functional consequences of non-histone lactylation

- MRE11 K673la: enhances DNA binding → promotes homologous recombination repair and chemoresistance (15)
- NBS1 K388la: stabilizes MRN complex → facilitates DNA repair (16)
- Snail1 K146/166la: induces nuclear translocation → activates EMT (18)
- Aldoa K147la: inhibits enzymatic activity → fine-tunes glycolytic flux (20)
- cGAS (multiple sites): blocks dsDNA binding → suppresses cGAS-STING innate immunity (22)

Compared with histone lactylation (global chromatin remodeling), non-histone lactylation provides rapid, protein-specific functional modulation. Their cooperation constitutes a multi-layered regulatory network driving tumor progression.

Writers, erasers, and readers of lysine lactylation

The dynamic and reversible nature of lysine lactylation (Kla) is governed by a tripartite enzyme system analogous to that of acetylation and phosphorylation, comprising “writers” (lactyltransferases), “erasers” (delactylases), and “readers” (effector proteins that recognize lactyl marks). This regulatory machinery enables cells to control the deposition, removal, and functional interpretation of lactyl groups in response to fluctuations in lactate concentration and metabolic status, thereby coupling glycolysis to gene expression and cellular signaling (**Table 1**).

**Table 1:** Writers, erasers, and readers of lysine lactylation.

Category	Representative proteins	Substrate specificity/target	Major function	Selected references
Writers	p300/CBP	Histone H3K18, H3K9; non-histone (PYCR1, NCL, MRE11, NBS1)	First identified lactyltransferases	Zhang et al., Nature, 2019 (4)
	HBO1	Histone H3K9	Catalyzes H3K9 lactylation	Galle et al., Genome Biol, 2022 (17)
	AARS1/AARS2	Global lysine lactylation (cGAS, BRD4, YAP)	Lactate sensors; suppress innate immunity	Li et al., Nature, 2024 (22); Zong et al., Nat Cell Biol, 2025 (28); Gao et al., Biomolecules, 2025 (23)
Erasers	HDAC1, HDAC2, HDAC3	Histone lysine residues (broad spectrum)	Major delactylases; also reverse activity	Moreno-Yruela et al., *Sci. Adv.*, 2022 (24)
	SIRT2	Histone lysine residues	NAD^+^-dependent delactylase	Zeng et al., *Cell Discov*, 2023 (29)
	SIRT3	Histone H3K9	Mitochondrial delactylase	Chen et al., *Mol Cell Proteomics*, 2025 (25)
	SIRT6	Histone lysine residues	NAD^+^-dependent histone delactylase	Nickel et al., *JBC*, 2024 (30)
Readers	DPF2	Histone H3K14la	Binds oncogenic gene promoters; drives transcription	Zhai et al., PNAS, 2024 (26)
	BRD4	BRD4 K317la (autoregulatory)	Inhibits degradation; promotes BET inhibitor resistance	Sun et al., 2025 (31)
	BRG1	Proposed lactylation reader	Executes downstream transcriptional programs	Shi et al., Front. Cell Dev. Biol., 2025 (32)

Writers. p300 and its paralog CBP were the first identified lactyltransferases. Overexpression of p300 elevates global histone Kla levels, whereas its silencing decreases them, establishing p300 as a functional writer of histone lactylation (4). Beyond p300/CBP, other histone acetyltransferases such as HBO1 have been reported to catalyze histone H3K9 lactylation (17). More recently, the alanyl-tRNA synthetases AARS1 and AARS2 were discovered to function as lactate sensors and global lysine lactyltransferases, directly binding lactate and catalyzing lactyl group transfer via a two-step mechanism involving lactyl-CoA (22). AARS1/2-driven lactylation has been implicated in suppressing cGAS-STING innate immunity and promoting tumor proliferation (23).

Erasers. Lactylation is reversible and can be actively removed by delactylases. Class I histone deacetylases (HDAC1, HDAC2, HDAC3) serve as major erasers of Kla, and they also exhibit reverse lactylation activity under certain conditions (24). Members of the sirtuin family, including SIRT2, SIRT3, and SIRT6, have been identified as histone delactylases. Notably, SIRT3 functions as an eraser of histone H3K9 lactylation and modulates transcription to inhibit esophageal cancer progression (25), whereas HDAC3 displays significantly higher delactylase activity than SIRT2, indicating context-dependent contributions.

**Figure 2:**
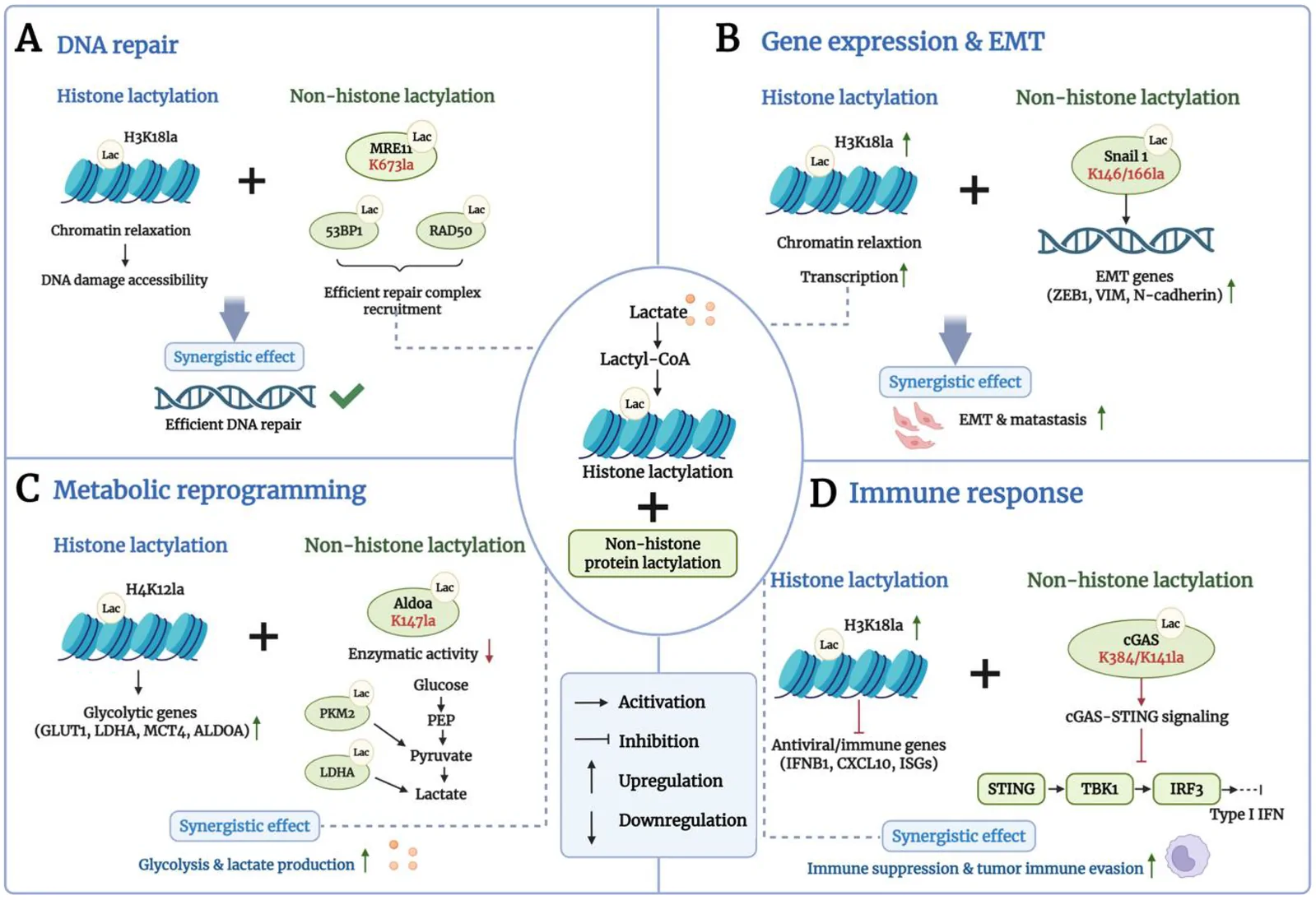
Synergistic network of histone and non-histone lactylation. Lactylation coordinately regulates four key cellular processes through modifications on both histones **(global chromatin remodeling)** and non-histone proteins (specific protein-function modulation). **(A)** DNA repair: Histone H3K18 lactylation (H3K18la) relaxes chromatin, facilitating access of repair factors; non-histone lactylation (e.g., MRE11 K673la) enhances homologous recombination repair. **(B)** Gene expression regulation and EMT: Histone lactylation (e.g., H3K18la, H4K12la) marks active promoters/enhancers; non-histone lactylation (e.g., Snail1 lactylation) induces nuclear translocation and drives epithelial-mesenchymal transition (EMT). **(C)** Metabolic reprogramming: Histone H4K12 lactylation activates transcription of glycolytic genes (GLUT1, LDHA, MCT4, ALDOA); non-histone lactylation (e.g., PKM2, ALDOA) fine-tunes enzyme activity to boost glycolysis. **(D)** Immune response: Histone lactylation upregulates antiviral/immune genes (e.g., IFNB1, CXCL10); non-histone lactylation (e.g., cGAS lactylation at multiple sites) suppresses STING-TBK1-IRF3 signaling, dampening type I IFN production. Created in BioRender. Tao, X. (2026). HYPERLINK "https://biorender.com/25t0tg3"https://BioRender.com/25t0tg3.

Readers. The identification of effector proteins that recognize lactylated lysine residues is an emerging area. DPF2 (double PHD fingers 2) has been characterized as a bona fide reader of histone H3K14 lactylation (H3K14la) (26). Using a multivalent photoaffinity probe, DPF2 was shown to bind H3K14la at promoters of oncogenic genes, and disrupting this interaction blunts cancer-related gene expression and cell survival. Additionally, BRD4, a well-known acetyl-lysine reader, may recognize lactylated lysine residues; BRD4 itself undergoes lactylation at K317 via AARS1, which inhibits its ubiquitination and degradation, promoting BET inhibitor resistance (Streptococcus anginosus-BRD4 study). The chromatin remodeling factor BRG1 has also been proposed as a lactylation reader (27). The known writers, erasers, and readers are summarized in.

(Sections II and III share **Figure 2**).

The lactylation axis in cancer: driving intrinsic malignancy and extrinsic immunosuppression

Lactylation levels are elevated in a cancer-type–specific manner. Analysis across multiple malignancies reveals that high lactylation is not universal but is particularly prominent in tumors with intense glycolytic activity, including glioblastoma, pancreatic ductal adenocarcinoma, hepatocellular carcinoma, and triple-negative breast cancer. This enrichment arises from tumor-intrinsic factors: constitutive activation of HIF-1α/2α driving LDHA expression, oncogenic mutations (e.g., KRAS, MYC) that potentiate the Warburg effect, and a lactate-rich hypoxic/acidic microenvironment. Importantly, lactylation acts as an active driver rather than a passive consequence of metabolic reprogramming. Direct inhibition of lactylation writers (e.g., p300, AARS1/2) suppresses tumor growth and restores anti-tumor immunity, confirming a causal role (15, 22). This synergistic regulatory network, while vital for normal physiology, is thus malignantly hijacked in the TME, where it couples metabolic reprogramming with immune evasion to fuel tumor progression.

### 2.1 Lactylation-driven metabolic reprogramming in tumors: the metabolism-epigenetics axis

Lactylation acts as a core molecular switch that directly links glycolysis to transcriptional control. This bidirectional “metabolism-epigenetics” crosstalk axis dynamically reshapes the metabolic phenotype of cancer cells, as detailed below.

#### 2.1.1 Histone lactylation – global activation of glycolytic genes

- Histone lactylation – Global Activation of Metabolic Genes: Zhang et al. first revealed that lactate from the TME is imported into cells via monocarboxylate transporters (e.g., MCT1) and, catalyzed by acetyltransferases like p300, covalently modifies histone lysine residues (e.g., H3K18, H3K9) to form Kla (4). This modification opens chromatin structure, significantly enhancing the accessibility of promoters for glycolytic genes (e.g., HK2, LDHA) while repressing genes involved in oxidative phosphorylation (e.g., PDK1), thereby pushing tumor cells toward a glycolytic phenotype (33). For instance, in non-small cell lung cancer, H3K18la modification at promoter regions upregulates HK-1 and SDHA expression, further strengthening the glycolytic flux (34). The specific upregulation of glycolytic genes by histone lactylation likely arises from two mechanisms: (i) promoters of glycolytic genes are enriched with HIF-1α and MYC binding motifs, which recruit p300 to these loci, enabling local H3K18 lactylation (34); and (ii) these promoters maintain an open chromatin state due to high H3K27ac levels, making them preferentially accessible to lactylation writers (17). Thus, histone lactylation acts as a positive feedback amplifier of glycolysis. Additionally, SRSF10 in tumor cells can enhance histone lactylation in macrophages via lactate, leading to activation of genes like CD206 and promoting an immunosuppressive TME (35).
- Non-histone lactylation – Precise Regulation of DNA Repair: The team led by Jian Yuan discovered that MRE11-K673 lactylation promotes homologous recombination (HR) repair by enhancing DNA-binding capacity, conferring resistance to platinum-based chemotherapy (15). Similarly, TIP60-mediated NBS1-K388 lactylation stabilizes the MRN complex and accelerates the repair of DNA double-strand breaks, leading to cisplatin resistance in gastric cancer (16). This synergy between histone-mediated metabolic activation and non-histone-mediated repair proficiency establishes a Metabolic Gene Activation-Repair Coupling.

#### 2.1.2 Non-histone lactylation – coupling metabolism, DNA repair, and resistance

In lenvatinib-resistant hepatocellular carcinoma, aberrant glycolytic activation causes lactate accumulation, which induces lactylation of the IGF2BP3 protein (IGF2BP3lac). This forms a metabolism-epigenetics axis: IGF2BP3lac stabilizes PCK2 and NRF2 mRNA, activating serine metabolism and elevating antioxidant capacity and the level of the methyl donor SAM. Increased SAM, in turn, drives m6A modification of PCK2/NRF2, forming a positive feedback loop (IGF2BP3lac-PCK2-SAM-m6A) that maintains high expression of resistance genes, thereby sustaining the drug-tolerant state (36). Similarly, tumor-derived lactate promotes the expression of the autophagy-enhancing protein RUBCNL via histone H3K18 lactylation (H3K18la), enhancing resistance to bevacizumab in colorectal cancer (37). Furthermore, glycometabolic reprogramming-induced XRCC1 lactylation confers therapy resistance in ALDH1A3-high glioblastoma (38). Under hypoxia, mitochondrial accumulation of AARS2 leads to lactylation-mediated inhibition of key oxidative phosphorylation enzymes, forcing tumor cells to rely on glycolysis for energy (39). Lactylation also promotes the activity of fatty acid synthase (FASN), enhancing lipid synthesis to support tumor cell membrane biogenesis and signal transduction (8). These examples illustrate how the metabolism-epigenetics axis drives phenotypes such as therapy resistance, immunosuppression via immune cell phenotypic changes, and altered metabolic pathways. These phenotypes, in turn, feedback to reinforce the metabolic reprogramming, creating a self-sustaining cycle.

The metabolic-epigenetic rewiring orchestrated by lactylation not only empowers cancer cells intrinsically but is also deeply coupled with immunosuppression. This coupling collectively establishes an immune-privileged niche, which represents another critical mechanism underpinning tumor progression.

### 2.2 Lactylation modifications facilitate immune evasion

Lactylation dynamically regulates tumor metabolic reprogramming via the metabolism-epigenetics axis. This process is deeply coupled with immune evasion, collectively establishing an immune-privileged microenvironment, which also represents another critical mechanism underpinning tumor proliferation and migration. Lactylation influences tumor immune escape through multiple mechanisms, including modulating immune cell functions and the expression of immune checkpoint molecules, thereby helping tumor cells evade immune attack. Furthermore, lactylation can alter the expression of cytokines and chemokines, further suppressing anti-tumor immune responses. Through the concerted action of these mechanisms, tumor cells can escape immune surveillance and sustain proliferation.

For instance, research from the team of Professor Long Zhang at Zhejiang University revealed that alanyl-tRNA synthetase AARS1/2 can function as a lactate sensor and lactyltransferase, directly binding lactate and catalyzing global lysine lactylation (22). This process involves two enzymatic steps: AARS1 and AARS2 act as lactyltransferases, catalyzing the transfer of lactate from a lactate donor (e.g., lactyl-CoA) to the ϵ-amino group (-NH_2_) of target lysine residues on proteins. Chemically, the carboxyl group (-COOH) of lactate forms an amide bond (-CONH-) with the lysine residue, generating lactyllysine (Kla). Specifically, AARS1/2-mediated lactylation modifies cyclic GMP-AMP synthase (cGAS) at key lysine residues (e.g., K50, K63, K198, K202). This modification does not affect cGAS’s enzymatic activity but specifically impairs its ability to bind to double-stranded DNA (dsDNA), the critical initial step for pathway activation. By blocking dsDNA recognition, lactylation effectively suppresses the cGAS-STING signaling cascade, thereby inhibiting innate immune surveillance and promoting immune evasion (22). This mechanism underscores the significant role of lactylation in immune evasion within the TME. In another example, tumor-derived lactate promotes the transcription of RUBCNL/Pacer via histone H3K18 lactylation (H3K18la). RUBCNL then interacts with BECN1 to promote autophagosome maturation, playing a key role in the proliferation and survival of hypoxic cancer cells (37).

#### 2.2.1 Reprogramming immune cell function to foster immunosuppression

**Figure 3:**
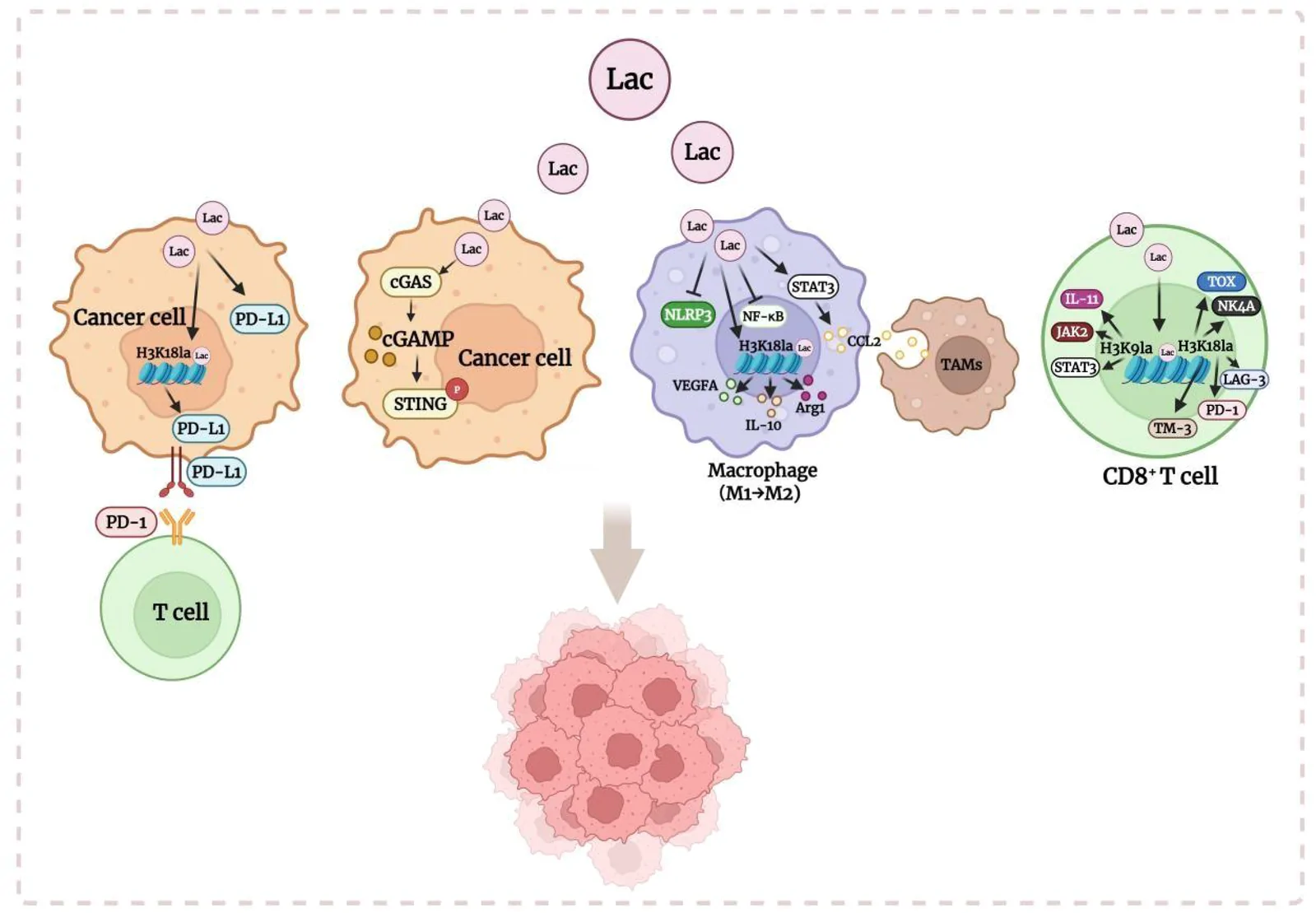
Lactate modulates the tumor immune microenvironment to drive immunosuppression. Created in BioRender. Yu, P. (2026). HYPERLINK "https://biorender.com/3upj71p"https://BioRender.com/3upj71p.

Lactate (Lac), derived from glycolytic tumor cells, exerts pleiotropic immunosuppressive effects on various immune cells within the tumor microenvironment (TME) (**Figure 3**). In CD8+ T cells, Lac influx induces histone H3K18 lactylation (H3K18la), which upregulates the expression of exhaustion-associated transcription factors (e.g., TOX, NR4A) and inhibitory receptors (e.g., PD-1, TIM-3, LAG-3), leading to T cell dysfunction. In macrophages, Lac promotes M2-like polarization (TAMs) by enhancing H3K18la, thereby activating signaling pathways (e.g., STAT3) and increasing the production of immunosuppressive (e.g., IL-10, VEGFA) and pro-tumorigenic factors (**Table 2**). In tumor cells,Lac can intrinsically upregulate immune checkpoint PD-L1 expression via H3K18la and modulate the cGAS-STING pathway. The concerted action of these mechanisms fosters an immunosuppressive TME that facilitates tumor immune evasion and progression. TAM, tumor-associated macrophage.

**Table 2:** Lactylation modulates immunosuppressive functions of myeloid cells in the tumor microenvironment.

Immune cell type	Reference	Mechanism (lactylation target)	Regulated gene/signaling pathway	Cancer type	Effect/function
Macrophages (MDMs)	Zhang,D. et al. (4)	Histone modification (H3K18)	Arg1, Vegfa	Breast cancer	Drives macrophage polarization from pro-inflammatory (M1) to anti-inflammatory (M2) phenotype.
Macrophages (MDMs)	Li, X.-M. et al. (40)	Histone modifications (H3K18)	RARγ(inhibition)	Colorectal cancer (CRC)	Inhibits RAR γ expression, inducing IL-6 production, which activates STAT3 in tumor cells, thereby promoting tumorigenesis
Macrophages (MDMs)	De Leo, A., et al (41)	Histone modifications(p300)	IL-10, TGF-β (transcription)	Glioblastoma	Activates transcription of immunosuppressive factors (e.g., IL-10, TGF-β), promoting the polarization of MDMs toward an immunosuppressive phenotype.
Macrophages (MDMs)	Gu, J., et al (42).	RIG-I lactylation	RIG-I lactylation → inhibits Nlrp3 → M2 polarization	colorectal cancer	Drives macrophage M2 polarization.
Macrophages (MDMs)	Sun, K., et al. (43)	Non-histone modification (ENSA-K63la)	ENSA-K63la → STAT3/CCL2 →TAM recruitment & immunosuppression	Pancreatic ductal adenocarci noma(PDAC)	Promotes therecruitment of tumor-associated macrophages (TAMs) into the TME.
VSIG4+Tumor-Associated Macrophages (TAMs)	Pan, Z., et al. (44)	Histone modification (H3K18)	VSIG4+ TAM-H3K18la→SPP1/STAT3→pro-tumorigenic interactions	Anaplastic thyroid cancer	Fosters pro-tumoral interactions between TAMs and neutrophils; blocking VSIG4 reduces lactate and restores CD8+ T cell function.
Tumor-Infiltrating Myeloid Cells (TIMs)	Xiong, J. et al. (45)	Non-histone modification (METTL3 K281, K345)	H3K18la→METT L3/m6A→JAK1/S TAT3 signaling	Colorectal cancer (CRC)	Induces immunosuppression and tumor immune evasion.
Macrophages (MDMs)	Sun, J., et al. (46)	Histone modification (H3K18)	CCL18	Ovarian cancer	Activates CCL18 expression, directly promoting tumorigenesis.

The studies listed below utilized macrophages derived from monocytes (MDMs) either through *in vitro* differentiation, ex vivo isolation from tumor tissues identified by surface markers (e.g., CD14+), or in specific transgenic models. Details of the cellular origin are confirmed in the corresponding primary publications.

The table focuses on macrophages; other myeloid cells are discussed in the main text.

In the tumor microenvironment (TME), lactylation-driven phenotypic remodeling of macrophages plays a critical role in facilitating immune evasion. Macrophages can differentiate into two distinct polarization states: classically activated “M1” macrophages, which are pro-inflammatory, and alternatively activated “M2” macrophages, which exhibit anti-inflammatory and immunosuppressive functions. Lactylation modifications drive the transition of macrophages from a pro-inflammatory (M1) to an anti-inflammatory (M2) phenotype, upregulating the expression of immunosuppressive factors like Arginase 1 (Arg1) and consequently inhibiting T cell activity (**Table 3**). Research from Filippo Veglia’s team at the Moffitt Cancer Center elucidated a mechanism by which high-glucose environments influence monocyte-derived macrophages (MDMs) via histone lactylation: aberrant glucose metabolism elevates lactate levels, which is then utilized by histone lactyltransferases like p300 to catalyze the covalent attachment of lactate to histone lysine residues. This modification alters chromatin architecture and activates the transcription of immunosuppressive factors such as IL-10 and TGF-β, thereby polarizing MDMs toward an immunosuppressive phenotype (41). This process may be further amplified by mitochondrial dynamics — recent findings indicate that mitochondrial fission enhances glycolytic flux, promoting lactate production. The accumulated lactate, in turn, directly upregulates Arg1 expression via histone H3K18 lactylation, accelerating M2 polarization (Redox Biol, 2024). Furthermore, tumor-derived lactate can suppress the transcription of the Retinoic Acid Receptor Gamma (RARγ) in macrophages through H3K18 lactylation. This leads to aberrant elevation of Interleukin-6 (IL-6) in the TME. IL-6 activates the STAT3 signaling pathway in colorectal cancer (CRC) cells, enhancing their proliferation and survival, while simultaneously augmenting the pro-tumoral functions of macrophages, thereby establishing a positive feedback loop (40). As a central metabolic cue in the TME, lactate remodels macrophage phenotype through multiple mechanisms. Studies show that tumor-derived lactate activates key signaling axes such as ERK/STAT3 (47), HIF-1α/2α (33), and Gpr132 (48), inducing a pro-tumoral M2 polarization, which subsequently suppresses anti-tumor immune responses. In summary, lactate integrates metabolic control, epigenetic modification, and signal pathway activation to multi-targetedly reprogram macrophage function, establishing itself as a core driver of tumor immune evasion. This aligns with the “lactate clock” concept proposed by Zhang D.’s team, which describes how endogenous lactate in bacteria-stimulated M1 macrophages drives a temporally programmed sequence of H3K18 lactylation-dependent gene expression, activating immunoregulatory genes such as Arg1 and IL-10 and promoting the transition from a pro-inflammatory M1 to an anti-inflammatory M2 phenotype (4). Notably, this epigenetic regulation exhibits tissue specificity. For instance, in the ovarian cancer microenvironment, macrophage H3K18 lactylation activates CCL18 expression, directly promoting tumor progression (46). Further research indicates a synergistic interaction between metabolic reprogramming and lactylation in tumor-associated macrophages (TAMs). Lactate influx via the monocarboxylate transporter MCT1 not only fuels oxidative phosphorylation and fatty acid oxidation after conversion to acetyl-CoA, providing an energy basis for M2 polarization, but also remodels chromatin accessibility through histone lactylation, promoting the transcriptional activation of M2 markers like Arg1 and IL-10, thereby forming a metabolic-epigenetic regulatory circuit (33). Additionally, lactate upregulates the expression of METTL3 in tumor-infiltrating myeloid cells (TIMs) via H3K18 lactylation. METTL3, in turn, enhances JAK1/STAT3 signaling phosphorylation through the m6A-YTHDF1 axis, reinforcing the immunosuppressive capacity of macrophages (45). Preclinical studies confirm that inhibiting histone lactylation reverses the immunosuppressive phenotype of TAMs in glioblastoma models, restoring anti-tumor immunity. Corroborating this, clinical sample analyses show a significant positive correlation between histone lactylation levels and macrophage immunosuppressive activity in GBM patients (41). It is important to note that lactylation exerts its effects not only directly via histone modification but also indirectly through signaling pathways, collaboratively reshaping macrophage function. For example, in colorectal cancer liver metastasis (CRLM), intratumoral Escherichia coli induces RIG-I lactylation, which disrupts the recruitment of NF- κ B to the Nlrp3 promoter, leading to transcriptional downregulation of Nlrp3. This impairs antigen presentation and promotes M2 polarization, ultimately inhibiting CD8+ T cell activity (42). In pancreatic ductal adenocarcinoma (PDAC), lactate produced by tumor cell glycolysis activates the STAT3/CCL2 signaling axis via ENSA-K63 lactylation (ENSA-K63la), recruiting TAMs and driving their immunosuppressive phenotype while inhibiting the infiltration of cytotoxic T cells (43). Research on anaplastic thyroid cancer (ATC) further reveals that VSIG4+ TAMs maintain STAT3-mediated SPP1 transcription through H3K18 lactylation (H3K18la), fostering pro-tumoral interactions between TAMs and neutrophils. Blocking VSIG4 reduces lactate levels and restores the anti-tumor function of CD8+ T cells (44). In conclusion, lactylation modifications, through both direct (histone modification) and indirect (signaling pathway) mechanisms, reprogram macrophage function and modulate T cell activity, establishing itself as a key hub for immune evasion across various cancer types.

**Table 3:** Lactylation modulates T cell function to foster an immunosuppressive TME.

Cell type	Reference	Mechanism (lactylation target)	Regulated gene/pathway	Cancer type	Effect/function
Regulator T cells	Watson, M. J. et al. (49)	Non-histone modifications (APOC2 K70)	APOC2	NSCLC	Generates FFAs, recruits Tregs, and contributes to immunotherapy resistance.
regulatory T cells	Gu, J., et al. (50),	Non-histone modificatio n (Moesin K72)	TGF-β/SMAD3 signaling	—	Lactate induces Moesin lactylation at K72, activating TGF-β/SMAD3 signaling to maintain Treg stability and suppressive function.
T regulator cell -Treg	Xue, Q., et al (51).	Histone modification (H3K18la)	TNFR2	Malignant Pleural Effusion	Lactate promotes H3K18la-dependent expression of TNFR2, enhancing the immunosuppressive function of Tregs and accelerating disease progression.
CD8^+^ T cell	Tong, H., et al (52).	Non-histone modification (PD-L1K72)	PD-L1	—	Attenuates lysosomal degradation of PD-L1, upregulating its surface level to help tumor cells evade CD8+ T cell attack.
CD8^+^ T cell	Raychaudhuri, D., et al (53).	Histone modifications (H3K18la, H3K9la)	—	—	Histone lactylation (H3K18la, H3K9la) impairs CD8^+^ T cell effector function.
CD8^+^ T cell	Wang, R., et al (54).	Histone modifications H3K9	IL-11/JAK2 /STAT3	Head and Neck Squamous Cell Carcinoma	H3K9la activates IL-11/JAK2/STAT3 signaling, upregulating immune checkpoint molecules and leading to CD8^+^ T cell dysfunction.
CD8+ T cell	Zhang, C.,et al (55)	Histone modification (H3K18la)	Immunosuppres sive molecules (CD39, CD73, CCR8)	Non-Small Cell Lung Cancer	H3K18la upregulates immunosuppressive molecules (e.g., CD39, CD73, CCR8), promoting Treg infiltration/function and creating an immunosuppressive TME that inhibits CAR-T cell activity.
CD8+T cell	Huang, Z.W., et al (56).	Histone lactylation (via STAT5)	STAT5-PD-L1	Acute Myeloid Leukemia	STAT5 activates glycolysis, increasing lactate and PD-L1 expression, inducing CD8+T cell exhaustion.
CD8+ T cell	Zhou, C., et al (57).	Histone lactylation (via PRMT3)	PRMT3-PD-L1	Multiple tumor models in female mice	Suppresses CD8+ T cell activity.
CD8+ T cell	Ma, Z., et al (58).	Histone modification (H3K18la on B7-H3 promoter)	B7-H3	Mouse tumor models	Inhibits CD8+ T cell infiltration and cytotoxicity.
CD8+ T cell	Ding, C.H., et al. (59)	Histone modification (H3K18la) via PRMT3	PRMT3-PD-L1	Hepatocellular Carcinoma	Enhances PD-L1 expression, inhibiting CD8+T cell function.
regulatory T cells	Sun, T., et (60).	Histone modificatio n H3K18la)	CD39,CD73	Glioblastoma	Upregulates CD39 and CD73 xpression, enhancing adenosine roduction to suppress effector T cells and romote Treg ctivation.
T-ALL ells	Wu, W., et al (61).	Histone modificatio ns H3K18la, 3K27ac)	IGFBP2,IARS	T-cell Acute Lymphoblastic eukemia T-ALL)	Affects T cell function, romoting immune escape.

information not explicitly reported in the cited study.

As core executors of adaptive immunity, T cells directly eliminate tumor cells via CD8+ cytotoxic T lymphocytes (CTLs) and coordinate anti-tumor immune responses through CD4+ helper T cells (e.g., Th1). However, aberrant metabolites in the tumor microenvironment (TME), particularly lactate, subvert T cell functional balance via epigenetic reprogramming. Lactate drives the expansion of immunosuppressive regulatory T cells (Tregs) while simultaneously inhibiting the activation and cytotoxic capacity of effector CD8+ T cells, ultimately leading to immune evasion.

Research demonstrates that lactate, through histone H3K18 lactylation (H3K18la), directly upregulates the expression of CD39, CD73, and CCR8 in Tregs, promoting adenosine generation and Treg tumor infiltration, thereby suppressing effector T cell activity (60). Concurrently, H3K18la enhances the immunosuppressive function of Tregs by activating TNFR2 transcription, accelerating the progression of malignant pleural effusion (51). Furthermore, lactylation of the MOESIN protein stabilizes the Treg phenotype by potentiating TGF-β receptor signaling (50).

In CD8+ T cells, lactate dynamically regulates gene promoter activity via H3K18la and H3K9 lactylation (H3K9la). H3K18la induces high PD-L1 expression on tumor cells by activating the POM121/MYC/PD-L1 axis, thereby inhibiting CD8+ T cell cytotoxicity (55), while H3K9la upregulates immune checkpoint molecules via the IL-11/JAK2/STAT3 signaling pathway, leading to CD8+ T cell dysfunction (53, 54). The metabolic-epigenetic crosstalk in CD8+ T cells is further evidenced by several axes: the STAT5-lactate-PD-L1 pathway promotes T cell exhaustion (56), circATXN7-dependent suppression of NF-κB signaling attenuates anti-tumor activity (57), the PRMT3-PDHK1-lactate axis enhances PD-L1 expression via H3K18la (59), and H3K18la modification of the B7-H3 promoter directly inhibits CD8+ T cell infiltration and cytotoxicity (58).

Clinical intervention strategies indicate that targeting lactate production (e.g., using LDHA inhibitors) or combining glycolytic inhibition with immune checkpoint blockade can reverse Treg-mediated immunosuppression and restore the anti-tumor activity of CD8+ T cells (55, 58, 60). In summary, lactate shapes the immunosuppressive phenotypes of T cells through multidimensional epigenetic reprogramming, providing a critical theoretical foundation for targeting the metabolic-immune axis of the TME.

Additionally, tumor cells can evade CD8+ T cell attack via GCN5-mediated lactylation of PD-L1, which inhibits its lysosomal degradation, leading to upregulated PD-L1 expression and conferring an indirect immune evasion advantage to the tumor cells (52).

Beyond directly suppressing immune cell function, lactate accumulation in the TME exacerbates immunosuppression through metabolic reprogramming. For instance, lactylation of PYCR1 in liver cancer cells enhances glycolysis, leading to excessive lactate accumulation that acidifies the microenvironment and inhibits CD8+ T cell activity, thereby promoting immune evasion (62).

Similarly, in T-cell acute lymphoblastic leukemia (T-ALL), the Warburg effect in tumor cells causes lactate overaccumulation, significantly elevating global H3K18la levels. Genome-wide analyses reveal that, compared to normal T cells, H3K18la sites in T-ALL cells undergo a dynamic redistribution from immune-related genes to pro-leukemic genes (e.g., IGFBP2, IARS), forming a transcriptionally active, super-enhancer-like pattern that directly drives the high expression of proliferation-related genes. Mechanistically, H3K18la acts synergistically with H3K27 acetylation (H3K27ac) to stabilize the oncogenic epigenetic program. Disruption of H3K18la significantly reverses the aberrant transcription of leukemia-associated genes. Clinical correlation analyses further confirm that high expression of IGFBP2 and IARS is closely associated with poor prognosis in T-ALL patients (61). This indirect reprogramming of the tumor cells’ own epigenetic state and the surrounding environment enables them to evade T cell attack, thereby creating substantial space for their proliferation and migration.

Lactylation in MDSCs and dendritic cells

In addition to macrophages and T cells, lactylation critically influences the immunosuppressive activity of myeloid-derived suppressor cells (MDSCs) and dendritic cells (DCs), two abundant myeloid populations within the TME.

MDSCs. MDSCs are major mediators of immune suppression in the TME. Lactate, produced abundantly via the Warburg effect, enhances MDSC immunosuppressive function through multiple lactylation-dependent mechanisms. One study reports that lactate induces histone lactylation, which upregulates TET2 expression. TET2 then, via STAT3, modulates ARG1 promoter methylation to increase ARG1 expression, thereby enhancing MDSC-mediated T cell suppression (63). Similarly, lactate has been shown to upregulate SGK1 expression in MDSCs via TET2-mediated demethylation, promoting immunosuppression in a lung cancer model (64). Beyond these epigenetic mechanisms, LDHA-driven lactate metabolism in prostate cancer promotes MDSC activation, and LDHA inhibition enhances anti-PD-L1 therapy (65). Furthermore, in microsatellite instability-high colorectal cancer, MNDA recruits EP300 to the CXCR2 promoter, mediating H3K18 lactylation to promote CXCR2 transcription and PMN-MDSC infiltration, driving PD-1 resistance (66). In pancreatic ductal adenocarcinoma, a high-LDHA TME increases monocytic MDSC numbers and elevates uPAR expression, further contributing to immunosuppression (67). Collectively, these studies establish lactylation as a key regulator of MDSC immunosuppressive function across multiple cancer types.

Dendritic cells. DCs are critical for initiating anti-tumor T cell responses. However, lactate accumulation in the TME subverts their function. Early studies showed that lactate conditions DCs to downregulate the trafficking protein VAMP3, impairing cross-presentation and antigen degradation (68). More recent work has uncovered specific lactylation-driven mechanisms. In colorectal cancer, downregulation of mitochondrial pyruvate carrier (MPC) leads to excessive lactate accumulation, which drives histone lactylation in DCs, repressing CD33 expression—a key marker of DC maturation—and thereby impairing CD8+ T cell activation while reducing anti-PD-1 efficacy (69). A lactate-SREBP2 signaling axis has also been shown to drive DCs toward a tolerogenic mregDC phenotype, promoting cancer progression. Most directly, in radioresistant tumors, lactate induces DNMT1 K1247 lactylation in DCs, enhancing its methyltransferase activity to silence STAT1 transcription via promoter hypermethylation, which suppresses MHC-I-dependent antigen presentation and CD8+ T cell activation (70, 71). These findings highlight that lactylation impairs DC function at multiple levels—from maturation and cross-presentation to the epigenetic silencing of MHC-I antigen presentation machinery.

Despite these advances, the roles of lactylation in MDSCs and DCs remain less explored compared with macrophages and T cells. Future studies are needed to dissect the specific lactylation “writers” and “erasers” operative in these cells, to identify additional lactylated non-histone targets, and to determine whether lactylation could be therapeutically targeted to restore DC function and inhibit MDSC-mediated immunosuppression.

### 2.3 Lactylation modifications drive tumor proliferation and metastasis

Having established lactylation as a pivotal regulator of immunosuppression, it is crucial to recognize that its pro-tumorigenic effects extend far beyond immune evasion. As a central mediator of tumor metabolism, lactylation directly remodels the epigenetic and signaling networks of tumor cells themselves, driving their malignant phenotype. Studies have shown that lactylation not only accelerates tumor proliferation by enhancing the activity of oncogenic signaling pathways (e.g., Wnt/β-catenin, PI3K/AKT) but also remodels the tumor microenvironment by modulating the function of metabolic enzymes (e.g., LDHA, PYCR1), thereby providing the energy and biosynthetic substrates required for invasion and metastasis. This metabolic-epigenetic synergy establishes lactylation as a key driver enabling tumor cells to overcome local constraints and achieve systemic dissemination. The following sections will systematically elaborate on the multi-dimensional regulatory mechanisms of lactylation in tumor proliferation and metastasis.

Lactylation modifications act synergistically with metabolic reprogramming to propel tumor malignancy. In breast cancer, KCNK1 activates LDHA to increase lactate production, which in turn promotes histone H3K18 lactylation (H3K18la). This establishes a lactate-LDHA-H3K18la positive feedback loop that activates downstream pro-metastatic genes (including LDHA itself) and enhances tumor invasiveness (4, 72). In intrahepatic cholangiocarcinoma (iCCA), Nucleolin (NCL) undergoes p300-catalyzed lactylation at lysine 477 (K477la) in glycolytically hyperactive cells. This modification promotes the efficient translation of MAPK Activating Death Domain Protein (MADD) by regulating RNA splicing, thereby activating the ERK signaling pathway and driving tumor proliferation and invasion (52) (73).

Beyond histones, lactylation of non-histone proteins plays a critical role. In liver cancer, p300-catalyzed lactylation of PYCR1 at K381 enhances its enzymatic activity, boosting proline synthesis and mitochondrial metabolism, which subsequently drives tumor growth and migration via the IRS1/PI3K/AKT signaling axis (50). Furthermore, lactylation of the transcriptional coactivator p300 itself at K1411 stabilizes the Wnt/β-catenin signaling pathway, upregulating the expression of oncogenes like c-Myc and Cyclin D1 to directly promote cell cycle progression (4). Proteomic analysis in cervical cancer identified lactylation of the non-histone protein PPP1R14B at K140; a decrease in this K140la modification was found to promote cancer cell proliferation and migration, and its reduced expression correlates with decreased CD8+ T cell infiltration and poor patient prognosis (53).

Clinical translational studies underscore the prognostic value of lactylation. In hepatocellular carcinoma (HCC), a prognostic model based on lactylation-related genes (LRGs) such as HDAC2 and SRRM1 effectively predicts an immunosuppressive microenvironment and recurrence risk (74). Similarly, in prostate cancer (PCa), a model built with LRGs like ALDOA and KIF2C is significantly associated with the enrichment of Tregs/M2 macrophages and chemotherapy resistance (75).

Collectively, these findings reveal that lactylation remodels the malignant tumor phenotype through a complex metabolism-epigenetics-immunity interaction network. Targeting lactylation modifications—for instance, by inhibiting LDHA or p300 activity—holds promise for blocking oncogenic signaling pathways and restoring therapeutic sensitivity, offering a novel strategic direction for interventions across multiple cancer types.

## 3 Emerging therapeutic directions

The elucidation of lactylation’s pro-tumorigenic mechanisms has unveiled a promising therapeutic landscape. Emerging strategies can be broadly categorized into several interconnected approaches: (i) targeting the source of lactate to cut off the substrate for lactylation (metabolic intervention); (ii) directly targeting the lactylation modification process itself using epigenetic tools (epigenetic intervention); (iii) counteracting the downstream immunosuppressive effects of lactylation, particularly through combination with immunotherapy; and (iv) leveraging novel delivery systems and biomarker-based stratification for personalized therapy. The following sections will detail the progress and potential of these strategies. As a pivotal hub connecting tumor metabolic reprogramming and epigenetic regulation, the elucidation of lactylation’s pro-tumorigenic mechanisms provides a novel perspective for overcoming the limitations of conventional therapies. Although chemotherapy, targeted therapy, and other interventions have achieved certain success, there remains an unmet clinical need for strategies specifically targeting the lactylation network. Recent research has focused on metabolic enzyme inhibitors (e.g., LDHA inhibitors), epigenetic editing tools (e.g., p300 lactylation antagonists), and combination strategies with immune checkpoint blockade. These approaches aim to multi-dimensionally intervene in lactate generation, lactylation modification, and downstream signaling, demonstrating dual potential for reversing the immunosuppressive microenvironment and enhancing treatment sensitivity. Direct evidence linking lactylation inhibition to tumor suppression. Several lines of causal evidence support that blocking lactylation per se inhibits cancer progression. In glioblastoma, pharmacological inhibition of the lactyl-CoA synthetase GTPSCS reduces H3K18la levels and suppresses tumor growth (11). Genetic ablation or small-molecule inhibition of p300 decreases histone lactylation and impairs xenograft proliferation (4). Mutation of specific lactylation sites (e.g., MRE11 K673, NBS1 K388) sensitizes tumors to chemotherapy and reduces DNA repair capacity (15, 16). These findings establish lactylation as a functionally required driver rather than a mere epiphenomenon. The following sections will systematically elaborate on these innovative therapeutic strategies targeting lactylation and their translational prospects.

### 3.1 Metabolic enzyme inhibition: cutting off the lactate source

Targeting key glycolytic enzymes represents a primary strategy for reversing lactylation modifications. LDHA inhibitors (e.g., Phenyllactic acid) suppress lactate production, thereby blocking NBS1 lactylation at K388, impairing homologous recombination repair capacity, and ultimately reversing chemotherapy resistance (16). In glioblastoma, the anti-epileptic drug Stiripentol inhibits LDHA/B activity, reduces H3K9la levels, restores temozolomide sensitivity, and possesses the ability to cross the blood-brain barrier (76). Specific inhibitors targeting the KCNK1/LDHA axis positive feedback loop in breast cancer can break the lactate-LDHA-H3K18la vicious cycle, inhibiting tumor invasion and metastasis (72). These metabolic interventions, by controlling lactate at its source, provide a universal therapeutic framework applicable across multiple cancer types.

### 3.2 Epigenetic editing: targeting the modification network

Developing small-molecule antagonists against the “writer” enzymes of lactylation and their effector proteins has become a hotspot in epigenetic therapy. p300 inhibitors, by blocking its lactylation at the K1411 site, inhibit the Wnt/β-catenin signaling pathway and downregulate the expression of oncogenes such as c-Myc (4). Clinical sample analysis in intrahepatic cholangiocarcinoma (iCCA) shows that high NCL-K477la levels are significantly associated with poor patient prognosis, suggesting its potential as a prognostic biomarker (73). Targeting the p300-mediated lactylation modification or blocking the NCL-MADD-ERK signaling axis effectively inhibits the growth of iCCA in xenograft models, offering a new therapeutic target for this refractory tumor. Furthermore, combining glycolysis inhibitors to intervene in metabolic reprogramming may synergistically enhance the anti-tumor efficacy of epigenetic therapies. Research in cervical cancer discovered that disrupting the interaction between DPF2 and H3K14la significantly suppresses oncogene transcription, providing a paradigm for targeting “reader” proteins (26). Such strategies enable reshaping of the tumor transcriptome at the epigenetic level.

### 3.3 Direct targeting of the lactylation machinery

Beyond indirect strategies that limit lactate availability, emerging efforts aim to directly inhibit the enzymatic machinery responsible for lactylation deposition. AARS1 and AARS2, recently identified as universal lactyltransferases that sense intracellular L-lactate and catalyze global lysine lactylation, represent attractive direct targets (22). The alanyl-tRNA synthetase domain of AARS1/2 provides a druggable pocket, and small-molecule inhibitors blocking their lactyltransferase activity could suppress global lactylation with potentially higher selectivity than general p300 inhibitors. Similarly, nuclear GTPSCS functions as a lactyl-CoA synthetase that generates lactyl-CoA within the nucleus to fuel histone lactylation, and its pharmacological inhibition has been shown to reduce H3K18la levels and suppress glioblastoma growth in preclinical models (11).

In parallel, proteolysis-targeting chimeras (PROTACs) that degrade lactylated oncoproteins or their upstream writers are beginning to emerge. A PROTAC targeting ACAT2, a lactylation-driven effector, has been developed in pancreatic cancer models (77), and advanced PROTAC technology has been applied to attenuate H3K18la levels and SNAI1 transcription in atherosclerosis (78), suggesting that PROTAC-based degradation of lactylation-related proteins could be adapted for cancer therapy (77).

Nevertheless, direct targeting of lactylation faces substantial challenges. Drugging p300/CBP must overcome concerns about off-target effects on acetylation, given that p300 is fundamentally an acetyltransferase. The identification of selective lactyltransferase inhibitors that spare canonical acetylation functions remains an unmet need. Additionally, the development of Kla-specific PROTACs requires potent, selective binders to the lactyl-lysine epitope, which is structurally similar to acetyl-lysine. Future efforts combining structure-guided drug design, high-throughput screening, and proteomic validation will be essential to translate these direct targeting strategies into clinical candidates.

### 3.4 Immune combination therapy: rebalancing the microenvironment

The close association between lactylation and the immunosuppressive microenvironment has spurred the development of novel combination therapies. In pancreatic cancer and hepatocellular carcinoma, the combination of lactate dehydrogenase inhibitors with anti-PD-1 antibodies can reverse Treg function, promote CD8+ T cell infiltration, and significantly enhance the efficacy of immune checkpoint blockade (ICB) (43, 50). Targeting ENSA-K63la or CCL2 can remodel the pancreatic cancer microenvironment and overcome ICB resistance (43). Furthermore, inhibiting cGAS lactylation activates the innate immune response, restoring dual surveillance against both viral infection and tumors (22). These strategies, which simultaneously target metabolic and immune pathways, provide a promising approach for immunotherapy in “cold” tumors.

### 3.5 Nanodelivery systems: synergistic multi-mechanism intervention

Novel nanocarriers enable multi-target synergistic effects by co-delivering metabolic inhibitors and epigenetic modulators. For instance, iPR@M1/Se nanoparticles (a selenium-based nanoplatform coated with M1 macrophage membrane) co-loaded with the glycolytic inhibitor Lonidamine and the PARP inhibitor Senaparib not only inhibit lactate production but also activate the cGAS/STING pathway, promoting dendritic cell maturation and T cell activation, thereby significantly suppressing the progression of triple-negative breast cancer (79). Similarly, liposome-delivered IGF2BP3 siRNA combined with 2-DG can block the lactylation-m6A positive feedback loop, reversing lenvatinib resistance in hepatocellular carcinoma (36). Such delivery systems offer spatiotemporally control, addressing the challenges of off-target effects and drug resistance associated with conventional monotherapies.

### 3.6 Clinical translation: from biomarkers to personalized therapy

Prognostic models based on lactylation-related genes (LRGs) are advancing the field toward personalized therapy. An 8-gene signature constructed in hepatocellular carcinoma can predict responses to immunotherapy and chemotherapy sensitivity (80), while a 5-LRG model in prostate cancer is significantly associated with Treg/M2 macrophage enrichment and poor prognosis (81). Pan-cancer lactylation scoring systems (e.g., for KIRC, LGG) assess immune microenvironment features, providing a cross-cancer tool for predicting ICB efficacy (82). These models, increasingly integrated with spatial transcriptomic and single-cell sequencing data, are progressively guiding clinical stratified treatment.

### 3.7 Challenges and future perspectives

Despite the promising potential of strategies targeting lactylation, their clinical translation faces challenges such as tissue specificity, off-target toxicity, and blood-brain barrier penetration.

Moreover, the tremendously high expression levels of lactate dehydrogenase (LDH) in many tumors require drug doses that are typically not clinically feasible, posing an additional hurdle for LDHA-targeted therapies(PMID: 36496155). Multi-modal therapies that combine metabolic reprogramming inhibitors, epigenetic editing tools, and immune activators may overcome the limitations of single-mechanism approaches through synergistic effects (11, 83). Accelerating the translation of mechanistic insights into clinical applications holds the promise of ushering in a new era of metabolism-epigenetics-immunity integrated therapy for solid tumors.

## 4 Conclusion

In summary, lactylation, as a novel post-translational modification mechanism bridging tumor metabolism and epigenetic regulation, provides a revised conceptual framework for understanding the tumor microenvironment, metabolic reprogramming, and immune evasion. This review has systematically deconstructed the multi-dimensional regulatory network of lactylation in malignant progression, revealing its molecular mechanisms in driving tumor cell proliferation, metastasis, and the establishment of an immune-privileged niche through the remodeling of the metabolism-immune axis. From the global impact of histone lactylation on chromatin architecture to the modulation of signaling pathways by non-histone lactylation, and further to the coordinated modification of metabolic enzymes and immune checkpoints, lactylation demonstrates a complex network that dynamically controls the tumor phenotype. Although innovative therapies targeting lactylation have shown promise in preclinical studies, challenges such as tissue specificity, blood-brain barrier penetration, and off-target toxicity remain to be addressed.

Looking forward, the integration of metabolomics, spatial transcriptomics, and single-cell multi-omics technologies will progressively clarify the dynamic landscape of lactylation in tumor heterogeneity and drug resistance mechanisms. AI-driven drug design platforms are expected to accelerate the development of targeted antagonists for lactylation “writer” and potential “eraser” enzymes. Concurrently, novel nanocarrier technologies will enable the spatiotemporally coordinated delivery of metabolic inhibitors and epigenetic modulators. Furthermore, pan-cancer lactylation scoring systems, combined with real-time monitoring techniques, are poised to advance the clinical application of lactylation modifications as liquid biopsy biomarkers. By integrating multi-dimensional strategies encompassing metabolic intervention, epigenetic remodeling, and immune activation, lactylation holds significant potential as a pivotal therapeutic target for overcoming current bottlenecks in cancer therapy. This approach offers a new paradigm for achieving a trinity of metabolism-epigenetics-immunity integrated treatment in the era of precision oncology.
